# Nanocarriers and macrophage interaction: from a potential hurdle to an alternative therapeutic strategy

**DOI:** 10.3762/bjnano.16.10

**Published:** 2025-01-31

**Authors:** Naths Grazia Sukubo, Paolo Bigini, Annalisa Morelli

**Affiliations:** 1 School of Medicine and Surgery, University of Milano-Bicocca, Via Cadore 48, Monza, Italy; 2 Department of Biochemistry and Molecular Pharmacology, Istituto di Ricerche Farmacologiche Mario Negri IRCCS, Via Mario Negri 2, Milano, Italy

**Keywords:** drug delivery, macrophages, nanomedicine, polarization, RNA-based therapies

## Abstract

In the coming decades, the development of nanocarriers (NCs) for targeted drug delivery will mark a significant advance in the field of pharmacology. NCs can improve drug solubility, ensure precise distribution, and enable passage across biological barriers. Despite these potential advantages, the interaction with many biological matrices, particularly with existing macrophages, must be considered. In this review, we will explore the dual role of macrophages in NC delivery, highlighting their physiological functions, the challenges posed by the mononuclear phagocyte system, and innovative strategies to exploit macrophage interactions for therapeutic advantage. Recent advancements in treating liver and lung diseases, particularly focusing on macrophage polarization and RNA-based therapies, have highlighted the potential developments in macrophage–NC interaction. Furthermore, we will delve into the intriguing potential of nanomedicine in neurology and traumatology, associated with macrophage interaction, and the exciting possibilities it holds for the future.

## Review

### 1 Introduction

In the vast nanomedicine landscape, the design and development of nanocarriers (NCs) for precise drug delivery are a pivotal innovation. NCs address significant pharmacological challenges, such as enhancing drug solubility, ensuring specific distribution, and facilitating the crossing of biological barriers [1]. Tailoring NCs to transport drugs, mRNAs, and other therapeutic agents directly to the site of pathology represents a significant advancement in medical treatment modalities. This approach can significantly support the shift towards more targeted and efficient therapeutic strategies. This progress results from decades of technological evolution, during which NCs have become indispensable components of drug delivery systems, known for their adaptability and efficiency [2].

The “family” of nanoparticles (NPs) includes a broad range of materials such as lipids, polymers, proteins, dextran, silica [3], and metals such as iron and gold [4–5]. Each material is chosen for its unique properties, such as size, hydrophilicity, and charge, that make it suitable for acting as a drug carrier. NCs can be functionalized on their surface to improve the stability and solubility of high-payload encapsulated cargos, promote transport across membranes, and extend circulation times. These advantages could reduce the negative effects of off-target drug accumulation and improve the release to the disease sites compared to current delivery systems [6].

Despite the expected applications in the biomedical field, the journey of NPs from research to clinical application faces significant hurdles, primarily due to interactions with the mononuclear phagocyte system (MPS). After administration in host bodies, NCs encounter systems of phagocytic cells, predominantly resident macrophages such as Kupffer cells (KCs) in the liver and macrophages in the spleen and lymph nodes, that sequester them. This occurs often independent of their design and structure [7]. Although a significant challenge, this interaction presents a unique clinical application opportunity.

Macrophages are involved in the pathogenesis of several diseases and thus can be considered a therapeutic target, exploiting their natural ability to phagocyte external agents such as NCs. Both monocytes and macrophages perpetuate tissue damage during chronic inflammatory disorders. They are implicated in preventing and resolving inflammation and wound-healing response [8]. Strategies for manipulating macrophage activation and function are diverse, ranging from depleting macrophages in diseased tissues, such as in cancer immunotherapy [9–11], to employing non-surgical treatments like extracorporeal shock wave therapy in various rheumatic diseases to promote resolution and healing [12].

The interplay between NCs and the immune system, especially macrophages, presents a complex scenario of challenges and insights pivotal for developing effective treatment options. Macrophages exhibit extraordinary versatility by adopting various functional phenotypes, such as classically activated (M1-like behavior) and alternatively activated (M2-like behavior) macrophages. M1 macrophages are involved in pro-inflammatory responses crucial for defending against pathogens. M2 macrophages mediate anti-inflammatory effects and may promote tumor growth and metastasis through their pro-tumor characteristics [13–14].

This dynamic and complex spectrum of macrophage activity features nuanced challenges and opportunities in leveraging macrophage responses to enhance the therapeutic potential of NCs. Recent research has highlighted the dual role of macrophages in the context of nanomedicine. While their ability to recognize and engulf NCs can impede the delivery of therapeutic agents to target tissues, it also opens avenues for novel strategies that exploit macrophage behavior for benefits, like targeted drug delivery and immunomodulation [2,7–8]. This review will explore the physiological functions of macrophages and the challenges of NC filtering by the MPS and conclude with innovative strategies to exploit these interactions for therapeutic benefit.

### 2 Physiological functions of macrophages

#### 2.1 Macrophage origin and functions

Macrophages are immune cells derived from the yolk sac, fetal liver in mice, or differentiated by circulating monocytes [15]. They act as the first line of defense in tissue by recognizing and engulfing pathogens and cellular debris via phagocytosis. This process is facilitated by detecting pathogen-associated molecular patterns (PAMPs) through pattern recognition receptors (PRRs) and the following degradation in lysosomes using hydrolytic enzymes and reactive oxygen species (ROS). Additionally, macrophages present antigen fragments through major histocompatibility complex (MHC) molecules, activating adaptive immune responses [16].

#### 2.2 Macrophage polarization

Macrophages are involved in tissue repair and homeostasis, regulating inflammation and its resolution by adopting different functional states, simplified in classically activated (M1) and alternatively activated (M2). This activation occurs on a spectrum, with various intermediate states influenced by microenvironmental signals. These states can exhibit overlapping functions and markers, demonstrating the plasticity and adaptability of macrophages in different physiological and pathological contexts. Within the M2 phenotype, macrophages can be further classified into four subgroups based on their specific activating stimuli, that is, M2a, M2b, M2c, and M2d [17]. Each subgroup plays distinct roles, such as tissue repair, immune regulation, or tumor progression, emphasizing the complexity of macrophage activation. While the M1/M2 classification provides a valuable framework for understanding macrophage polarization, it does not encompass the full complexity of macrophage biology [18]. Further research is needed to explore the diverse activation states of macrophages and their specific roles in health and disease. Therefore, in this review, we will utilize the commonly used M1/M2 dichotomy.

#### 2.3 Macrophage activation: essential mechanisms in disease and healing

The role of macrophages in disease pathogenesis is closely tied to their activation states.

**M1 macrophages – drivers of inflammation:** M1 macrophages are induced by pro-inflammatory stimuli like lipopolysaccharide (LPS) and interferon gamma (IFN-γ), which trigger a strong pro-inflammatory response. These cells release cytokines such as tumor necrosis factor-α (TNF-α), interleukin (IL)-6, and IL-1β, essential for pathogen clearance and initiating immune defense mechanisms [19]. However, if an inflammation remains active for extended periods, it can contribute to tissue damage and chronic inflammation. This prolonged M1 activity is a hallmark of diseases like rheumatoid arthritis (RA) and inflammatory bowel disease (IBD).

The activation of M1 macrophages is primarily mediated by the nuclear factor-κB (NF-κB), which is triggered by microbial ligands binding to toll-like receptor 4 (TLR4) [19]. This interaction leads to the expression and release of type-1 interferons (IFN-α and IFN-β), which further amplify the immune response through the Janus kinase/signal transducers and activator of transcription (JAK/STAT) signaling cascade [18–19].

**M2 macrophages – pro-resolving functions:** In contrast, M2 macrophages are activated by anti-inflammatory stimuli, such as IL-4 and IL-13, and are primarily involved in resolving inflammation and promoting tissue repair. They secrete anti-inflammatory cytokines such as IL-10 and transforming growth factor β (TGF-β) [20], as well as vascular endothelial growth factor (VEGF) and platelet-derived growth factor (PDGF), which promote extracellular matrix (ECM) deposition and angiogenesis, playing a key role in conditions like fibrosis and wound healing [14,17].

The M2 activation state is mediated by the STAT6 pathway via the IL-4Rα1 receptor, triggered by the binding with IL-4 or IL-13. This process inhibits the M1 response by blocking NF-κB and activator protein 1 (AP-1) by activating the peroxisome proliferators-activated receptor-γ (PPARγ) [17].

Within this broader M2 category, macrophages can be further classified into four subtypes, that is, M2a, M2b, M2c, and M2d, based on their specific activation signals, cytokine production, and functional roles. The already mentioned M2 macrophages driven by IL-4 and IL-13, are known as M2a. M2b macrophages, induced by immune complexes and TLR ligands, regulate inflammation through the secretion of IL-10 and low levels of TNF-α. M2c macrophages, stimulated by IL-10 and glucocorticoids, resolve inflammation and promote tissue regeneration. Last, M2d macrophages, known as tumor-associated macrophages (TAMs), are activated by adenosine and IL-6 and characterized by their pro-angiogenic role. They produce VEGF and matrix metalloproteinases (MMPs), which are particularly relevant in tumor progression [17,21–22].

**Therapeutic implications of macrophage polarization:** Based on the aforementioned mechanisms, it is evident that macrophage dysregulation can be implicated in various clinical conditions. For instance, TAMs, which often exhibit an M2-like phenotype, contribute to tumor progression by promoting angiogenesis and suppressing antitumor immune responses [22]. Reprogramming these macrophages towards an M1 phenotype has shown promise in improving antitumor immunity and enhancing cancer immunotherapy outcomes. Additionally, natural compounds such as berberine and quercetin can modulate macrophage polarization by inhibiting M1 pathways or promoting M2 activity, highlighting the therapeutic potential of targeting macrophage states in inflammatory and degenerative diseases [23–24].

### 3 NC accumulation in macrophages: a challenge for drug delivery

To exert their therapeutic effects, NCs must efficiently reach and accumulate in the target tissues. However, numerous biological barriers hinder this process, which vary depending on the administration route, as well as the type and stage of the patient’s disease, thus restricting precise delivery [1].

#### 3.1 Systemic administration and biological barriers challenges

One of the primary challenges of NC therapy is overcoming biological barriers following systemic administration, which remains the most common delivery route despite the potential advantages of local approaches [6]. Once in the bloodstream, NCs are exposed to a wide range of forces, such as fluid shear stress, blood flow, opsonization, excretion, and interaction with the MPS, all of which influence NC stability and delivery. This challenging and complex scenario is furthermore amplified when NCs are administered upon pathological conditions, especially in inflamed tissues where immune cells such as macrophages are highly activated (as described in Section 2) [25]. Interestingly, studies have shown that both macrophage phenotypes significantly affect NP internalization. They actively engulf NCs and accelerate their clearance, acting differentially in a time-dependent manner and altering the fate of nanomaterials [26].

In addition to immune-related barriers, the physicochemical properties of the nanomaterial itself can impair the NCs’ ability to protect their cargo, promote extravasation, and reach the target tissue effectively. Fluid forces can strip NCs of their surface coatings, reducing their ability to adhere to vessel walls, an essential step for extravasation into the parenchyma of target tissues. Particles larger than 200 nm and shapes like ellipsoids, discoids, and nanorods with higher aspect ratios are more effectively localized close to blood vessel walls, enhancing their internalization into endothelial cells and potentially improving their therapeutic delivery [1].

After systemic administration, NCs tend to accumulate in hepatic tissue because of its large blood volume, supplied by both the portal vein and hepatic artery. This allows the NCs to extravasate towards the liver’s sinusoidal capillary walls, where they interact with various hepatic cells, particularly KCs and mononuclear phagocytic cells. These cells, which account for 80–90% of the body’s total macrophage population, play a central role in immune surveillance and clearance of foreign entities. KCs utilize scavenger receptors (SRs), a superfamily of transmembrane glycoproteins expressed by myeloid cells, to detect and eliminate unwanted substances such as NPs, pathogens, and oxidized lipoproteins [27–28]. These SRs function also as PRRs identifying both endogenous (e.g., damaged cells) and exogenous molecules (e.g., pathogens), activating intracellular signal transduction, and maintaining hepatic homeostatic functions [29]. Notably, SR-A1, expressed in KCs, is essential for clearing infections caused by the Gram-positive bacterium *Listeria monocytogenes* [30].

In nanomedicine, SRs are also responsible for clearing negatively charged NPs such as those composed of silica [31–32]. This interaction leads to the internalization of NCs via endocytosis, reinforcing the role of the MPS in NC clearance.

#### 3.2 Role of the mononuclear phagocyte system

Initially classified as a distinct cell lineage, the MPS consists of phagocytic cells, predominantly monocytes, and macrophages [33] that can rapidly sequester NCs after injection. This clearance process begins with opsonization in the bloodstream, mediated by opsonins that recognize plasma proteins (serum albumin, apolipoproteins, complement components, and immunoglobulins) adsorbed onto the surface of circulating NPs. This forms the so-called “protein corona” (PC), a layer of more than 300 proteins that effectively masks the functionalization of groups coated on the NC surface. The formation of this corona acts as a clearance signal, prompting macrophages to recognize and engulf NCs [34]. The denser the proteins adsorbed onto the NC surface, the faster the uptake into the liver and spleen [35–36].

Several factors influence PC formation and NC clearance, including NP size, surface charge, hydrophobicity, surface chemistry, and the encountered biological fluid [37–39]. NPs larger than 200 nm in size tend to accumulate in the liver and spleen, while those with a diameter of less than 10 nm are rapidly eliminated by the kidneys as they can pass through renal filtration mechanisms. Surface charge also plays a crucial role in NC clearance; cationic NCs are preferable for penetrating cells. However, they are rapidly cleared by macrophages via SR-mediated pathways. In contrast, neutral or slightly negatively charged NCs have longer half-lives in circulation but may face more hurdles in crossing biological membranes [1,6]. Table 1 summarizes the overall properties, in terms of bio-nano interactions, described among the different materials.

**Table 1:** Summary of NC types and their physicochemical properties.

NC Type	Material composition	Size range (nm)	Limitations	Solutions	Advantages	Ref.

Inorganic						

silica nanoparticles	silica (SiO_2_)	10–200	rapid clearance by the MPS	coating with hydrophilic polymers, functionalization	high surface area, customizable pore size	[3]
iron oxide nanoparticles	iron oxide (Fe_3_O_4_, Fe_2_O_3_)	10–100	agglomeration and recognition by MPS	surface coating with biocompatible materials, functionalization	superparamagnetic properties, good for imaging and therapy	[4]
gold nanoparticles	gold (Au)	1–100	rapid clearance by the MPS and potential cytotoxicity	PEGylation, functionalization	inert, easily functionalized, optical properties	[5]

Lipidic						

liposomes	phospholipids, cholesterol	50–200	rapid clearance by the MPS	PEGylation, functionalization	biocompatible, versatile, can encapsulate hydrophilic and lipophilic drugs	[9]

Polymeric						

chitosan nanoparticles	chitosan (natural polysaccharide)	50–300	limited stability and solubility	surface functionalization, drug conjugation	biodegradable, low toxicity, mucoadhesive properties	[40]
PLGA nanoparticles	poly(lactic-*co*-glycolic acid)	50–500	rapid clearance by the MPS and potential toxicity	PEGylation, functionalization	biodegradable, customizable release profiles	[41]

### 4 Strategies to enhance NC drug delivery by modulating macrophage uptake and enabling the endosomal escape

To improve the efficacy of NC-based drug delivery systems, it is crucial to develop strategies that reduce macrophage uptake and extend NC circulation time. This could be achieved by acting on NCs exploiting alternative administration routes or physicochemical modifications, or directly on the macrophages with immune evasion techniques.

#### 4.1 Alternative routes of administration

As discussed in Section 3.1, systemic administration remains the most common route in nanomedicine. However, undesired macrophage clearance is minimized through alternative delivery methods. Intranasal delivery has emerged as a promising strategy for targeting the central nervous system by bypassing the blood–brain barrier (BBB). This approach was demonstrated by the nose-to-brain administration of D6-cholestrol-loaded liposomes, which led to an accumulation of D6-cholesterol in the brain in healthy mice and in a murine model of Huntington's disease [42]. Similarly, inhalation for lung targeting [43], subcutaneous injection for reaching lymph nodes [44], or oral administration for gastrointestinal tract disorders [45] are important alternative routes.

#### 4.2 Macrophage depletion and modulation

Another approach to reducing macrophage uptake of NCs is to modulate their activity, thereby decreasing their overall presence in the target organs. KCs play a role in maintaining an inflammatory state in various liver disorders. Clodronate, a bisphosphonate, interferes with cell metabolism by inhibiting ADP/ATP translocase in the mitochondria, ultimately leading to KCs apoptosis. When encapsulated in liposome in combination with nintedanib, a triple tyrosine kinase inhibitor, there is also a reduction in the secretion of inflammatory cytokines, which enhances the antifibrotic effects. This has been demonstrated by Ji and colleagues in a mouse model of carbon tetrachloride (CCl_4_)-induced fibrosis, where they inhibited the proliferation of fibroblasts [46]. An alternative to depletion is the inhibition of KCs through chloroquine, an antimalaria agent that inhibits macrophage-specific endocytosis, or saturation of the uptake with other non-toxic NPs administered as a pre-treatment. The disruption of lysosome and endocytotic processes causes the engulfment of KCs and the temporary blockade of the MPS. Although the induced modulation of innate immunity is effective, these strategies may lead to an increased susceptibility to infection, toxicity, and other liver disorders because of the suppression of essential physiological roles of KCs [47–48].

#### 4.3 Surface masking and the “stealth” effect

At a subcellular level, the interaction between liver macrophages and NCs can be prevented by masking the NC surface with hydrophilic polymers such as PEG. PEGylation is widely used for its “stealth” effect, hindering protein adsorption on the hydrophobic polymer surface by steric repulsion [36]. However, the long-term use of PEGylated NCs for treating chronic diseases can lead to side effects, such as activation of the complement system, due to the accumulation of PEG in the body as a non-biodegradable polymer. Therefore, PEGylation must be carefully considered when designing NC-based therapies [49–50].

A strategy to avoid possible immunoreactions is to mask NPs by marking them as “self” and biomimetic. Coating NCs with membranes from red blood cells or neutrophils or decorating them with peptides can camouflage the NCs and prevent macrophage ingestion [51–52].

#### 4.4 Endosomal escape

After reaching the target site, as discussed in the previous paragraphs, NCs should release their cargo to exert their final effect. Endocytosis poses a significant challenge for delivering drugs and nucleic acids to the cytosol as most remain trapped in endosomes and subsequently degrade. Efficient delivery requires the payload to be released before lysosomal maturation, a crucial stage known as endosomal escape [53]. NCs enhance the delivery of biological therapeutics, and endosomal escape can be controlled by tuning their structure and physicochemical properties. For example, NCs designed with “proton sponge” capability contain materials that adsorb and buffer protons under acidic conditions and, typically, have high buffering capacities in the acidic pH range of endosomes (pH 5–6). Lipid nanoparticles (LNPs), which include cationic and ionizable materials, exhibit such intracellularly triggered delivery mechanisms and are often used to carry nucleic acids into cells. In this case, the endosomal escape is influenced by the molar ratio between ionizable lipids and mRNA nucleotides; thus, it protects the nucleic acid and promotes efficient in vivo delivery [54].

In summary, combining physicochemical modifications, surface coatings, and immune evasion techniques can significantly enhance the therapeutic potential of NCs by reducing macrophage uptake and extending circulation time. These strategies pave the way for more effective and targeted drug delivery systems.

### 5 Innovative therapeutic strategies using NCs

Recent advancements in NC technologies have highlighted their remarkable potential in targeting macrophages for therapeutic applications, capitalizing on the unique characteristics of these immune cells. Macrophages are highly versatile agents for drug delivery because of their ability to evade immune surveillance, perform phagocytosis, and home to inflamed or diseased tissues [14]. Additionally, their large size (≈25 μm) facilitates the efficient loading of diverse drugs (e.g., hydrophilic or hydrophobic) [55]. As detailed in Section 2, macrophages persist throughout acute and chronic inflammatory phases, broadening their therapeutic applicability across various pathological conditions.

This chapter examines strategies that position macrophages as direct biological targets of NPs, aiming to modulate their activity as a therapeutic intervention for various pathological conditions, rather than merely using them as biomimetic drug carriers.

#### 5.1 Targeted drug delivery to macrophages

As described previously, NCs provide an innovative approach for delivering therapeutics to macrophages, particularly by targeting specific subsets, such as M2-like macrophages in tumor microenvironments. One widely studied approach involves sugars like mannose and hyaluronic acid, which naturally bind to macrophage-specific receptors. Mannose-decorated NCs, for example, leverage the overexpression of mannose receptors (CD206) on polarized M2 cells. Hatami et al. demonstrated the efficacy of self-assembling Pluronic^®^ F127 polymer and tannic acid cores decorated with mannose in enhancing macrophage uptake [56]. The unique physicochemical properties of these mannose-decorated hybrid NPs, such as controlled particle size (≈265 nm) and stability ensured by negative zeta potential, make them highly effective for receptor-mediated endocytosis and intracellular drug delivery [57]. Similarly, hyaluronic acid-coated NCs target CD44 receptors on macrophages, improving drug delivery in inflammatory disease models and highlighting their potential in treating chronic inflammation and autoimmune conditions [58].

Beyond surface modifications, innovative strategies have used macrophages as “Trojan horse”-like carriers for drug-loaded NPs into injured or diseased sites. This strategy takes advantage of the macrophages’ innate ability to infiltrate diseased tissues, including hypoxic tumor regions that are otherwise difficult to access. Evans et al. demonstrated that macrophages loaded with hypoxia-activated prodrug NPs, such as tirapazamine (TPZ), significantly enhanced drug accumulation in hypoxic regions of solid tumors. Macrophage-mediated delivery achieved a 3.7-fold greater tumor weight reduction than free TPZ alone or in NP form [59]. This method underscores the dual utility of macrophages as both therapeutic targets and delivery vehicles.

Antibody-functionalized NCs are a powerful method for selective drug delivery. For example, anti-CD163 antibodies can be conjugated to NCs to target M2 macrophages specifically. This strategy has shown significant promise in modulating TAMs by delivering agents that either reprogram or deplete these cells, effectively inhibiting tumor growth and metastasis. Such selective targeting reduces off-target effects and minimizes damage to healthy tissues [60].

While these strategies highlight the versatility of NCs, their clinical translation requires careful attention to safety and sterility. For instance, endotoxins or LPS remaining on the NC surface after synthesis can enhance macrophage uptake but may also cause unwanted immune activation [61]. Consequently, ensuring sterility is a critical prerequisite for developing nanomedical devices.

#### 5.2 Harnessing macrophage plasticity for immunomodulation

The modulation of macrophage polarization has shown significant potential in addressing specific conditions. By leveraging the plasticity of macrophages, these approaches aim to dynamically repolarize macrophages between a pro-inflammatory M1 state and an anti-inflammatory M2 state, depending on the context. In cancer therapy, reprogramming M2 macrophages into M1 enhances antitumor immunity, while in chronic inflammatory diseases, shifting from M1 to M2 facilitates inflammation resolution and tissue repair. This modulation can be achieved using small molecules, cytokines, or nanotechnology to target key signaling pathways [62].

**M1 polarization in cancer therapy:** Inducing or sustaining M1 polarization has proven effective in enhancing antitumor immunity. For example, chimeric antigen receptor macrophages (CAR-Ms), engineered by Klichinsky et al., sustained a robust M1 phenotype for over 40 days, secreting pro-inflammatory cytokines that reprogrammed surrounding M2 macrophages into M1-like cells. This approach not only eliminated tumor cells but also enhanced the activation of antitumor T-cells [63].

Huo et al. further explored the impact of M1 polarization on CAR-Ms in models of HER2-positive ovarian cancer and lung metastases. They demonstrated that pre-polarizing CAR-Ms to an M1 phenotype significantly enhanced their antitumor efficacy, in vitro and in vivo, using murine models of intraperitoneal ovarian cancer and lung metastases. M1-polarized CAR-Ms reduced tumor burden, prolonged survival, and improved immune responses by increasing secretion of pro-inflammatory cytokines such as IL-12 and TNF-α [64]. These findings highlight the enhanced therapeutic potential of combining M1 polarization with CAR-M therapy, particularly in treating solid tumors with a challenging immunosuppressive microenvironment.

An innovative approach involves “tail-flipping” nanoliposomes incorporating carboxylated phospholipids, such as 1-palmitoyl-2-azelaoyl-*sn*-glycero-3-phosphocholine (PAPC), designed to selectively target M2 TAMs via scavenger receptors, such as SR-B1. When loaded with therapeutic agents such as the STAT6 inhibitor AS1517499, zoledronic acid, or muramyl tripeptide (MTP), these liposomes inhibited M2 polarization. In preclinical breast cancer models, PAPC nanoliposomes reduced tumor growth, inhibited the M2 phenotype, and prevented pre-metastatic niche formation, achieving up to a 70% reduction in tumor burden without inducing toxicity [65].

**M2 polarization in restoring inflammatory diseases:** Polarizing macrophages to the M2 phenotype has shown considerable promise in treating inflammatory diseases such as IBD and RA. M2 macrophages secrete anti-inflammatory cytokines like IL-10 and TGF-β, aiding inflammation resolution and tissue repair.

In IBD, sustaining the M2 phenotype is particularly challenging because of the pro-ferroptotic microenvironment, which undermines macrophage survival. Zhao et al. addressed this issue by developing calcium carbonate (CaCO_3_)-mineralized liposomes (CLF) loaded with the ferroptosis inhibitor Fer-1. These liposomes promoted M2 polarization via the CaSR/AKT/β-catenin pathway while protecting macrophages from ferroptosis. In murine models of IBD, CLF reduced oxidative stress, inhibited ferroptosis, and restored intestinal homeostasis by increasing the M2/M1 macrophage ratio [66].

In RA, the predominance of M1 macrophages in inflamed joints drives synovitis and cartilage destruction. Yang and colleagues developed folic acid-modified silver nanoparticles (FA-AgNPs) to target M1 macrophages via folate receptor-mediated endocytosis. Once internalized, these NPs scavenged ROS, induced M1 apoptosis, and facilitated M1 to M2 polarization. In murine models of collagen-induced arthritis, FA-AgNPs significantly reduced joint swelling, improved cartilage integrity, and outperformed standard treatments like methotrexate [67].

Stabilizing macrophage polarization for long-lasting therapeutic effects remains a significant challenge, as their activation states are dynamic and often characterized by mixed or transitional phenotypes. This underscores the need for refined approaches to enhance efficacy while minimizing risks such as excessive immune activation. RNA-based therapeutics offer a promising solution by precisely targeting genes that regulate macrophage polarization, paving the way for more adaptable treatments.

#### 5.3 RNA-based therapeutics in macrophage polarization

Developing RNA-based therapies that target macrophage polarization presents significant challenges, particularly in ensuring specific and compelling modulation without causing adverse side effects. However, therapeutic molecules such as miRNAs, siRNAs, and mRNAs show promising potential in modulating macrophage behavior.

**MicroRNA (miRNA) therapeutics:** miRNAs are small non-coding RNA molecules (≈22 nucleotides) that regulate gene expression post-transcriptionally by binding to the 3′-UTR of target mRNAs, thereby inhibiting their translation. Their ability to promote specific macrophage phenotypes has made miRNAs a powerful tool for macrophage modulation. For instance, miR-155 promotes the M1 phenotype by suppressing anti-inflammatory pathways, while miR-146a enhances endotoxin tolerance by modulating TLR signaling through Notch1 inhibition [68–69]. miR-221-3p drives M2 macrophages towards the M1 phenotype by inhibiting the JAK3/STAT3 signaling pathway. Conversely, miR-1246 promotes M2 polarization by targeting TERF2IP, activating STAT3, and inhibiting NF-κB [70–71].

Innovative delivery systems for miRNAs have further advanced their therapeutic application. Liu et al. developed a hybrid nanovector with dual redox/pH-responsive properties for targeted miRNA delivery in cancer therapy. This system, comprising galactose-functionalized polypeptides (GLC) coated with PEG-PLL copolymers (sPEG), was designed to release miR-155 specifically in the acidic tumor microenvironment. At neutral pH, the sPEG coating masked the cationic core, minimizing off-target effects, while at acidic pH, the coating detached, exposing GLC and enhancing miRNA uptake by TAMs. This nanovector, sPEG/GLC/155, increased miR-155 expression in TAMs 100–400-fold, leading to a robust M1-like polarization characterized by upregulated IL-12, iNOS, and MHC II, along with reduced M2 markers such as Arg1 and Msr2. The therapy also stimulated the activation of T-cells and natural killer cells, resulting in significant tumor regression [72].

**Small interfering RNA (siRNA) therapeutics:** siRNAs are double-stranded RNA molecules that induce the degradation of specific mRNAs, effectively silencing their expression. This approach has shown promise in mitigating inflammation and promoting tumoricidal macrophage polarization.

For inflammatory diseases like IBD, targeting pro-inflammatory cytokines with siRNA has proven effective. Laroui et al. developed polymeric NPs made of poly(lactic acid)–poly(ethylene glycol) block copolymer (PLA-PEG) grafted with the Fab’ fragment of F4/80 antibodies for specific macrophage targeting. These Fab’-bearing NPs delivered TNF-α siRNA to the colonic macrophages of mice, attenuating colitis more efficiently than non-targeted systems [73].

In cancer therapy, siRNAs have been used to reprogram TAMs to the M1 phenotype. For example, the co-delivery of a STAT6 inhibitor and IKKβ siRNA successfully repolarized M2-like TAMs into M1-like macrophages, enhancing antitumor immunity while minimizing immune side effects [74]. To improve targeting specificity, micellar nanodrugs with pH-sheddable PEG coronas have been designed, allowing the encapsulated siRNA and inhibitors to selectively act on M2 TAMs in the acidic tumor microenvironment. This strategy effectively suppressed tumor growth and metastasis, while avoiding off-target macrophage repolarization in non-tumor tissues, enhancing both safety and efficacy [74].

**Messenger RNA (mRNA) therapeutics:** mRNA-based therapies enable the expression of therapeutic proteins within macrophages, providing a versatile approach to modulate their polarization. mRNAs encoding anti-inflammatory cytokines, such as IL-10, have been used to promote M2 polarization, facilitating tissue repair and inflammation resolution in autoimmune diseases.

For instance, LNPs containing IL-10 mRNA, formulated with the ionizable amino lipid Dlin-MC3-DMA (MC3) and other lipids like DSPC, cholesterol, and DMG-PEG2000, have demonstrated significant efficacy in inducing the M2 phenotype, reducing inflammation and tissue damage in RA models [75]. Beyond cytokines, mRNA therapies have targeted macrophage-specific markers to induce phenotype switching. Polyethylenimine NPs grafted with mannose ligands have been used to deliver genes encoding CD163, a hallmark of M2 macrophages. This system successfully converted pro-inflammatory M1 macrophages into M2 macrophages in vitro, enhancing the release of anti-inflammatory cytokines and potentially mitigating inflammatory responses in vivo [76].

Transcription factors are another promising target for mRNA-based therapies. Delivering mRNAs encoding transcription factors such as PPARγ has shown the potential to promote M2 polarization, thereby supporting tissue regeneration and reducing chronic inflammation. These systems demonstrate the versatility of mRNA-based therapies in addressing various pathological conditions.

However, translating RNA-based therapeutics into clinical practice requires overcoming key challenges, such as ensuring molecular stability, achieving targeted delivery, and minimizing immune activation. Ongoing research is addressing these barriers by advancing RNA delivery systems [77]. For example, mesoporous silica NPs have been utilized to co-deliver miRNAs and small-molecule drugs to macrophages, successfully reprogramming TAMs in cancer models to favor antitumor immunity [78–79].

Recent breakthroughs in mRNA delivery technologies, exemplified by the success of the Moderna and Pfizer-BioNTech COVID-19 vaccines, provide a clear pathway for improving mRNA-based therapies. These vaccines use LNPs designed to facilitate endosomal escape, preventing RNA degradation within lysosomes and improving intracellular delivery efficacy [80–81].

Developing targeted delivery systems, summarized in Table 2, that can fine-tune macrophage activation, is a pivotal step in translating the therapeutic potential of RNA molecules into practical clinical applications, providing innovative solutions to the intricate challenges of nanomedicine.

**Table 2:** Targeting strategies and approaches to modulate M1/M2 macrophages.

Category	Type of NP	Functionalization	Strategy of Action	Ref.

Targeted drug delivery	NPs-mannose-decorated hybrid particles	mannose functionalization	targets CD206 on M2 macrophages for receptor-mediated endocytosis	[56–57]
	NPs-hyaluronic acid	hyaluronic acid coating	targets CD44 expressed by macrophages	[58]
	NPs-anti-CD163	anti-CD163 antibody	selective drug delivery to M2 macrophages	[60]
	hypoxia-activated prodrug-loaded macrophages	macrophages	“Trojan horse”-like delivery into hypoxic tumor microenvironments	[59]

Immunomodulation	“tail-flipping” PAPC nanoliposomes	carboxylated phospholipids (PAPC)	repolarizes M2 TAMs via SR-B1 scavenger receptors	[65]
	CaCO_3_-mineralized liposomes (CLF)	mineralized surface with ferroptosis inhibitor Fer-1	promoted M2 polarization via CaSR/AKT/β-catenin pathway and protected macrophages from ferroptosis	[66]
	folic acid-modified silver NPs (FA-AgNPs)	folic acid ligand on the surface	targets M1 macrophages via folate receptors, scavenging ROS and facilitating M1-to-M2 polarization	[67]

RNA-based therapeutics	NPs-miR-155	dual-responsive PEG-coating with galactose-functionalized polypeptides	promotion of M1 phenotype by suppressing anti-inflammatory pathways	[72]
	NPs-miR-146a	neutral polymeric carriers	promotes M2 phenotype by modulating TLR signaling through Notch1 inhibition	[69]
	NPs-miR-1246	Encapsulation of miR-1246	promotes M2 polarization by activating STAT3 and inhibiting NF-κB	[71]
	polymeric NPs-siRNA	Fab’-modified surface (F4/80 antibodies)	suppresses TNF-α in M1 macrophages to reduce inflammation, particularly in IBD models	[73]
	nanodrug STAT6 inhibitor S1517499-IKKβ siRNA	pH-sensitive PEG corona	repolarization of TAMs from M2 to M1 macrophages in acidic tumor microenvironments	[74]
	ionizable amino lipid Dlin-MC3-DMA (MC3)	LNPs formulated with ionizable lipids	delivery of IL-10 for M2 phenotype promotion	[75]
	polyethylenimine NPs	mannose ligands on the surface	converts M1 macrophages to M2 by delivering genes encoding anti-inflammatory markers like CD163	[76]
	mesoporous silica NPs	co-loaded with miRNAs and small molecules	modulates TAMs to favor antitumor immunity	[78–79]

### 6 Applications in liver and lung disorders

**Figure 1:**
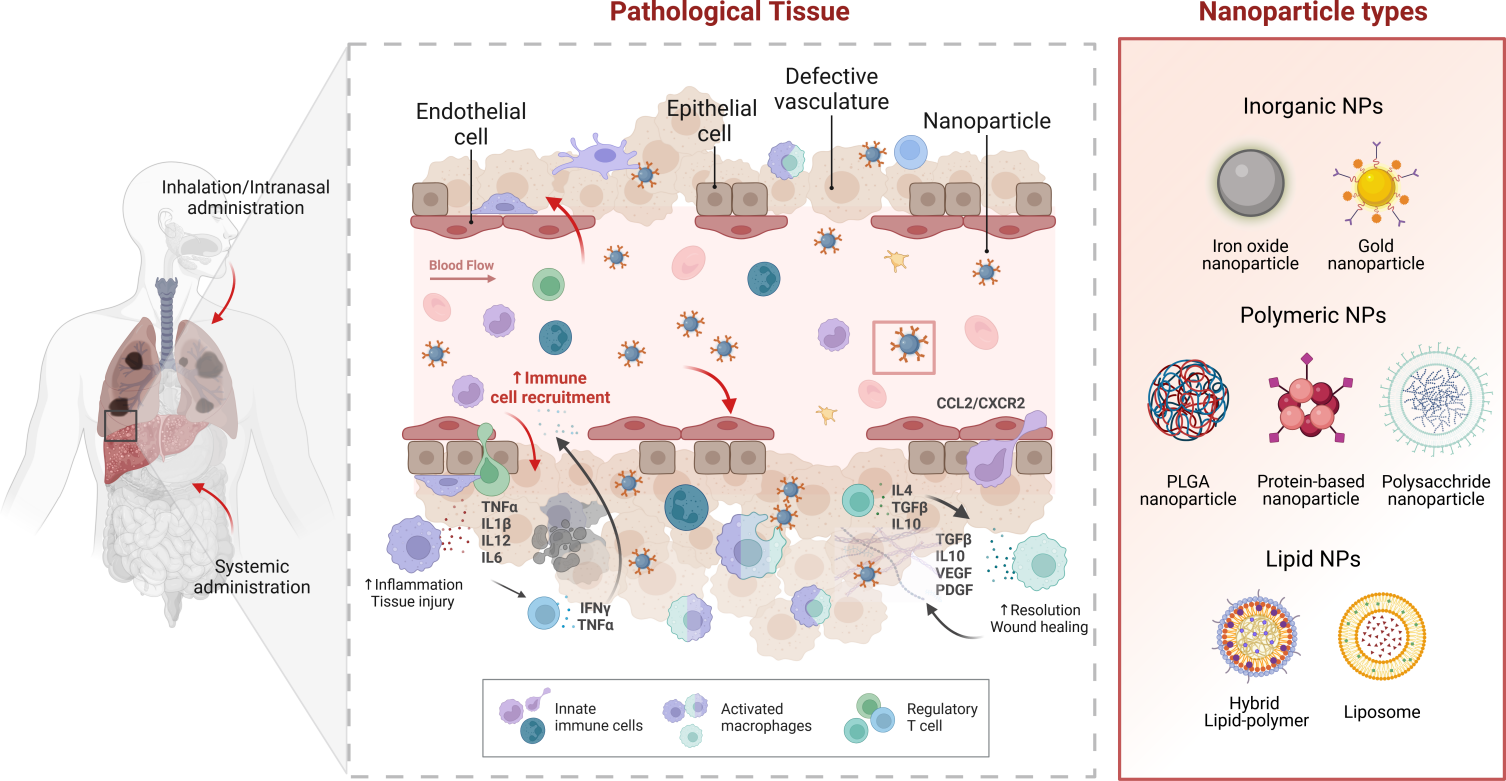
Nanoparticle targeting in liver and lung pathologies. Illustration of NC targeting in pathological tissues, specifically the liver and lungs. The diagram highlights administration methods, including inhalation/intranasal and systemic routes. It depicts how NCs interact within diseased tissues, showing their ability to modulate macrophage phenotypes. Inflammatory signals like TNF-α, IL-1β, and IL-6 increase tissue injury, while anti-inflammatory signals like IL-4 and TGFβ promote healing and resolution. NCs can influence these processes by targeting and modifying macrophage activity. The various types of NPs used in treatments are shown, including inorganic (iron oxide, gold), polymeric (PLGA, protein-based, polysaccharide), and lipid NPs (lipid-polymer hybrids, liposomes). Created in BioRender. Bigini, P. (2025) https://BioRender.com/f64r030. This content is subject to CC BY 4.0.

NCs can be used to treat liver and lung diseases. Their ability to enhance drug delivery, improve bioavailability, and target specific cells makes them valuable tools for managing conditions affecting these organs. Figure 1 illustrates these approaches within a pathological context.

#### 6.1 Targeting liver diseases

The liver, a key organ in metabolic processes, is an ideal target for NC-based therapies because of its unique anatomy, clearance functions, and resident KCs. As mentioned in Section 3.1, it plays a pivotal role in detoxification, protein synthesis, and regulating various biochemical pathways, making it a critical focus for therapeutic intervention. Hepatic macrophages constitute approximately 90% of the body’s total macrophage population and play important roles in liver health and disease. Leveraging this knowledge can improve treatment effectiveness while reducing side effects [28].

Liver diseases, including hepatitis, cirrhosis, and hepatocellular carcinoma (HCC), are major global health concerns, affecting millions worldwide and placing a significant burden on healthcare systems. Viral hepatitis, for example, affects over 300 million people globally and remains a leading cause of liver-related morbidity and mortality [82]. Cirrhosis, a long-term consequence of chronic liver fibrosis, leads to impaired liver function. HCC, the most common form of primary liver cancer, is often associated with underlying liver conditions like hepatitis and cirrhosis [82]. By exploiting the liver’s unique vascular structure and the natural propensity of KCs to phagocytose foreign particles, NCs can be designed to selectively administer antiviral drugs, antifibrotic agents, and chemotherapeutics [83].

**6.1.1 Hepatitis:** Hepatitis is an inflammation of the liver with various etiologies, including viral infections (hepatitis B and C), alcohol abuse, autoimmune diseases, and exposure to toxins. Among these, viral hepatitis is the most prevalent form, affecting 71 million people worldwide with hepatitis C alone, while hepatitis B causes approximately 887,000 deaths annually, being a considerable public health challenge, according to the World Health Organization (WHO) [82,84]. Effective management of hepatitis is critical because of its potential to progress to chronic liver disease, cirrhosis, and HCC if left untreated.

One approach to improve hepatitis treatment involves PEGylated liposomes loaded with antiviral drugs such as tenofovir, which can significantly reduce viral loads in hepatocyte cultures. Clinical studies have demonstrated ameliorated therapeutic outcomes and minimized systemic side effects [85–86]. Additionally, targeting KCs with mannose- or galactose-coated NPs enhances the delivery of immunomodulatory agents [87]. Mannose and galactose receptors, part of the SR family, facilitate the selective uptake of these NPs by KCs, helping to control inflammation and reduce liver damage.

**6.1.2 Liver fibrosis:** Liver fibrosis is characterized by the excessive production of ECM proteins, leading to scarring and impaired liver function. It often results from chronic liver injury due to various causes, including hepatitis (such as hepatitis B and C), long-term alcohol abuse, and non-alcoholic steatohepatitis, which is associated with obesity and metabolic syndrome. The progression of liver fibrosis is driven by the activation of hepatic stellate cells, which transform into myofibroblasts responsible for producing collagen and other ECM components. This fibrogenic response is significantly influenced by the interplay among a diverse population of immune cells, particularly macrophages. Chronic inflammatory responses sustain this process, with various damaging mediators, including ROS and other oxidative stress-related compounds, playing a critical role in perpetuating fibrosis [88].

It has been found by Beljaars et al., that both M1- and M2-like macrophage subsets coexist in fibrotic lesions in both human and mouse livers, highlighting their essential roles in the fibrotic process [89]. Therefore, targeting these macrophage populations with specific inhibitors can effectively mitigate liver fibrosis.

For instance, liposomes loaded with valsartan have been shown to significantly reduce fibrogenic cytokine levels by targeting specific receptors involved in fibrosis on hepatic stellate cells [90]. Another example includes fluorescent dexamethasone (Dex)-loaded liposomes, which have been demonstrated to deplete hepatic and systemic T cells and polarize macrophages towards an anti-inflammatory phenotype [91].

Polymeric NCs modulate collagen production, effectively reducing collagen type I deposition and mitigating fibrosis. Additional nanomaterials such as superparamagnetic iron oxide nanoparticles (SPIONs) and chitosan-based NPs are engineered with liver-cell-specific ligands like lactose or galactose, enhancing their specificity for treating liver fibrosis and HCC [92–93]. NCs co-loaded with clodronate and nintedanib have reduced fibrosis by depleting or inhibiting KCs, thus decreasing pro-fibrogenic activity [46]. These targeted approaches enhance precision, minimize off-target effects, and improve treatment efficacy.

Scavenger receptors expressed on KCs have also been targeted using molecules like phosphatidylserine (PS), which serves as a recognition signal for the phagocytosis of apoptotic cells. In a study by Wang et al., PS-modified lipid carriers loaded with curcumin (Cur–mNLCs) demonstrated improved retention time, bioavailability, and delivery effectiveness of the therapeutic agent, while also showing a reduction in liver damage and fibrosis in an in vivo CCl_4_-induced rat model [94].

Regarding gene therapy, NPs deliver siRNA or miRNA targeting key signaling pathways involved in fibrogenesis. For instance, lipoplex-based transfection has been utilized for macrophage targeting, wherein glucan-encapsulating sphingosine 1-phosphate receptor 2 (S1PR2)-siRNA NCs can attenuate hepatic inflammation and fibrosis. This is achieved by reducing the activation of the NLRP3 inflammasome in mouse models of bile duct ligation (BDL), methionine-choline-deficient high-fat diet (MCDHF), and CCl_4_-induced fibrosis [95].

The application of nanotechnology in liver fibrosis treatment holds great potential. It offers targeted delivery systems that enhance the efficacy of therapeutic agents while minimizing systemic side effects.

**6.1.3 Hepatocellular carcinoma (HCC):** HCC is the most common type of primary liver cancer and represents a significant global health burden [96]. The development of HCC is a complex, multistep process that involves genetic mutations, epigenetic alterations, and changes in the liver microenvironment. The prognosis for HCC is generally poor, mainly because of late diagnosis, limited treatment options, and high recurrence rates after treatment. Effective management of HCC requires a multifaceted approach, including surgical resection, liver transplantation, locoregional therapies such as radiofrequency ablation and transarterial chemoembolization (TACE), and systemic therapies. However, achieving targeted delivery to the tumor site is often difficult because of the limitations posed by the side effects of these treatments [97].

Recent advancements in nanotechnology have opened new avenues for the targeted delivery of chemotherapeutic agents and gene therapies to HCC cells, aiming to improve treatment efficacy while minimizing adverse effects. NPs can be engineered to recognize and bind to specific molecules overexpressed on the surface of HCC cells, such as glypican-3 (GPC3), a proteoglycan highly expressed in HCC but not in normal liver tissue [98]. This targeted approach allows for the selective delivery of therapeutic agents to cancer cells, sparing healthy cells and reducing systemic toxicity. For example, gold NPs functionalized with antibodies against GPC3 have been used in photothermal therapy to selectively kill cancer cells upon near-infrared light exposure, significantly reducing tumor size with minimal damage to surrounding tissues [99]. Moreover, NCs can be designed to deliver immunomodulatory agents, such as checkpoint inhibitors or cytokines, directly to the tumor microenvironment to enhance antitumor immunity. For example, NPs loaded with anti-PD-L1 antibodies can block the interaction between PD-1 on T cells and PD-L1 on tumor cells, strengthening T cells to attack HCC cells more effectively both in vitro and in vivo [100].

Overall, NPs offer an alternative solution for treating HCC by improving drug delivery precision and enhancing the antitumor immune response, potentially leading to better patient outcomes.

**6.1.4 Autoimmune hepatitis (AIH):** Autoimmune hepatitis (AIH) is a chronic inflammatory liver disease characterized by immune-mediated hepatocyte damage. This condition occurs when the immune system erroneously targets liver cells, causing inflammation that can lead to liver failure if not properly managed. AIH can affect individuals of any age, gender, or ethnicity, though it is more prevalent in females and typically presents during adolescence or middle age [101]. The pathogenesis of AIH involves a complex interplay of genetic predispositions, environmental triggers, and immune system dysregulation. Patients with AIH often present non-specific symptoms such as fatigue, jaundice, abdominal pain, and hepatomegaly, making early diagnosis challenging. Laboratory findings commonly include elevated liver enzymes, hypergammaglobulinemia, and the presence of autoantibodies such as antinuclear antibodies, smooth muscle antibodies (SMA), and liver/kidney microsomal antibodies (LKM-1) [102].

To dampen the immune response, traditional AIH treatments typically involve immunosuppressive therapies, such as corticosteroids and azathioprine. Although these treatments help control inflammation and prevent disease progression, they often come with substantial side effects, including an increased risk of infections, bone density loss, diabetes, and hypertension. Recent advancements in AIH treatment have focused on improving therapeutic efficacy while minimizing side effects. One such innovative approach, as demonstrated by Violatto et al., involves the conjugation of Dex to biodegradable Avidin-Nucleic-Acid-Nano-Assemblies (ANANAS). This method leverages the natural liver tropism of these nano-assemblies to enhance the targeting of therapeutic agents to the liver, thereby improving the treatment efficacy and reducing systemic side effects. Preclinical studies show that ANANAS-Dex significantly reduce liver inflammation and damage by efficiently targeting hepatic macrophages, offering a more focused and effective AIH treatment. This approach not only improves therapeutic outcomes but also mitigates adverse effects related to prolonged steroid use [103]. Future clinical trials will be crucial to validate this innovative therapy’s efficacy and safety and determine its potential role in the standard treatment regimen for AIH.

#### 6.2 Targeting lung diseases

The immune system plays an essential role in the lungs, representing the largest surface exposed to the external environment. Pathogens can enter through the inhaled air via the epithelial layer or through the bloodstream via the endothelium. Both circulating and resident immune cells are ready to be recruited in response to infection or injury. Alveolar macrophages (AMs) and interstitial macrophages (IMs) act as phagocytic sentinels, fulfilling homeostatic, metabolic, and repair functions [104–105]. They are involved in the onset of various lung diseases, such as asthma, chronic obstructive pulmonary disease (COPD), fibrosis, infections, and lung cancer [106]. The specific disease manifestation depends on its activation state and the microenvironment, as described in Section 2. These diseases often have fatal outcomes, and no effective treatment currently exists to restore lung function, representing the top leading causes of mortality alongside cardiovascular diseases. To address these challenges, NP-based delivery systems could offer advantages. Various preclinical and clinical studies are investigating the use of immunomodulatory agents to modulate macrophage activation and function. Their functional plasticity and complex involvement in the pathogenesis make them attractive targets for therapeutic intervention. The pulmonary route of administration is particularly significant as it allows for the direct delivery of drugs to the lungs for both local and systemic therapeutic needs [107–108].

**6.2.1 Obstructive lung diseases:** Obstructive lung diseases such as asthma and COPD are marked by restricted airflow due to partial or complete blockage of the airways. Asthma, a chronic inflammatory condition, is characterized by wheezing, chest tightness, shortness of breath, and coughing. COPD, usually caused by inhaling harmful particles like cigarette smoke, leads to ongoing and worsening airflow restriction [109]. Both conditions involve chronic inflammation, oxidative stress, and airway remodeling, causing structural and functional impairments [110]. M1 macrophages contribute to the inflammatory response by releasing pro-inflammatory mediators, worsening tissue damage and remodeling. An imbalance between M1 and M2 macrophages may lead to chronic inflammation and tissue destruction [111].

Conventional treatment is usually limited by low drug penetration. NCs can overcome this hurdle by offering new avenues for drug delivery that enhance effectiveness and reduce side effects.

AMs play a critical role in asthma by maintaining inflammation and tissue damage. Holotomography was used to analyze the morphological and ultrastructural changes in macrophages, providing nanoscale insights into inflammation. The efficiency of gold NP (AuNP)-loaded macrophages as a targeted delivery system was tested in an ovalbumin-induced asthma mouse model. Findings showed significant macrophage–NP interactions, highlighting the potential of macrophage-based systems for nanomedical applications and immunotherapeutic strategies in asthma treatment [112].

Researchers have developed an inhalable formulation of Roflumilast using lipid–polymer hybrid nanoparticles (LPHNPs) to improve its delivery to the lungs and specifically target AMs in COPD. Roflumilast, a phosphodiesterase-4 inhibitor, is known for its anti-inflammatory properties but causes significant side effects when taken orally. Delivering the drug directly to the lungs through inhalation can minimize these side effects while improving its therapeutic efficacy by targeting AMs. The LPHNPs consist of a polymeric core coated with a phospholipid layer enabling the loading of multiple agents. Moreover, Craparo et al. have demonstrated their effectiveness in COPD treatment in preliminary in vitro studies incorporating a mannose-conjugated ligand targeting CD206 receptors on AMs [113].

**6.2.2 Interstitial lung diseases:** Interstitial lung diseases are a group of lung disorders characterized by inflammation and scarring of the interstitial tissue in the lung parenchyma [114]. Pulmonary fibrosis is the progressive and irreversible accumulation of collagen and other fibrous proteins, leading to decreased lung function and respiratory failure, sustained by M2-like macrophages [115].

One approach for treating idiopathic pulmonary fibrosis (IPF) is the development of aerosolizable microgels (aeroμGel) that contain nintedanib-PLGA NPs and pirfenidone-liposomes. The aeroμGel with a size of approx. 12 μm resisted phagocytosis by AMs in vitro and in vivo and protected the entrapped drugs. The two nanoformulations effectively treated bleomycin-induced mouse model of pulmonary fibrosis. They resulted in reduced fibrosis progression, restored normal lung function, deactivated myofibroblasts, inhibited the progression of TGF-β, and suppressed the production of ECM components such as collagen I and α-SMA while extending the duration of their presence in the lungs. The increased local availability of both nintedanib and pirfenidone was due to the evasion of AM phagocytosis and prolonged lung retention with reduced systemic distribution [116].

An alternative strategy could be the direct targeting of lung macrophages, given their essential role in the pathogenesis and their natural ability to phagocyte foreign material. The study conducted by Codullo and colleagues investigates the use of Imatinib-loaded gold NPs (GNP-HCIm) functionalized with anti-CD44 for treating systemic sclerosis-related interstitial lung disease (SSc-ILD). In vitro tests revealed that GNP-HCIm inhibited the proliferation and induced apoptosis in lung fibroblasts. Additionally, they reduced IL-8 release, viability, and M2 polarization in AMs from SSc-ILD patients*.* In the bleomycin-induced mouse model of lung fibrosis, intratracheal administration of GNP-HCIm significantly limited collagen deposition. This suggests a promising therapeutic approach for SSc-ILD through local administration of targeted NPs [117].

In pathological lung conditions like IPF, alveolar epithelial cells and fibroblasts secrete SDF-1, attracting CXCR4-expressing cells (e.g., fibrocytes and macrophages). The CXCR4/SDF-1 axis is vital for tissue regeneration, influencing fibroblast proliferation and fibrosis development. Blocking CXCR4 with AMD3100 reduces fibrocyte migration. In addition, high plasminogen activator inhibitor-1 (PAI-1) levels, regulated by TGF-β, correlate with increased macrophage infiltration, fibrosis, and mortality. This study evaluates a treatment combining CXCR4 antagonism and PAI-1 inhibition using perfluorocarbon (PFC) emulsion polyplexes loaded with a fluorinated CXCR4 antagonist (F-PAMD) and siRNA. In vitro and in vivo tests showed high uptake, reduced fibrosis markers, prolonged lung retention, and therapeutic efficacy in acute lung injury (ALI) and IPF models, suggesting that this approach is a promising strategy for treating these conditions [118].

These innovative NP-based strategies offer targeted and efficient means to treat lung fibrosis, potentially improving outcomes for patients suffering from this debilitating condition. Moreover, these findings highlight the potential of intranasal and intratracheal delivery routes for IPF treatment using nanoformulations, exploiting the large surface of absorption of the pulmonary tissue [119–120].

**6.2.3 Infectious lung diseases:** Infectious diseases are a significant cause of illness and death worldwide. They can be divided by the causative agents in bacterial, parasitic, fungal, and viral infections, leading to the inflammatory clinical syndrome of pneumonia. However, the intracellular survival of pathogens, such as *Mycobacterium tuberculosis*, is often linked to the ability of certain strains to manipulate macrophage activation states, leading to granuloma formation and tissue damage [121].

As an antibacterial action, a new nanosystem (M33-NS) obtained by capturing SET-M33, a non-natural antimicrobial peptide, was evaluated for its efficacy in bacteria cells and mouse and rat models of pneumonia. M33-NS was effective against *Pseudomonas aeruginosa*, and the lung residence time of the antimicrobial peptide, administered via aerosol in healthy rats, was markedly improved by peptide functionalization of the NPs [122]. Bioinspired microrobots capable of moving in biological fluids represent another solution against *P. aeruginosa*. These NCs are antibiotic-loaded neutrophil membrane-coated polymeric NPs attached to natural microalgae for the active delivery of antibiotics in the lungs. In a mouse model of acute *P. aeruginosa* pneumonia, the microrobots effectively reduce bacterial burden and substantially lessened animal mortality, showing fast speed in lung fluid, uniform distribution, and low clearance by AMs upon intratracheal administration [123].

Nanodecoy systems, which utilize viral receptor analogs assembled onto fluid lipid-based membranes of nano or extracellular vesicles or NCs, offer a potential new tool to complement traditional therapeutic or preventive antiviral approaches. A semisynthetic self-assembling SARS-CoV-2 nanodecoy was developed by multimerizing the biotin-labeled virus-cell receptor -ACE2- ectodomain onto ANANAS. In vivo studies in mice demonstrated that the nanodecoy’s biodistribution and safety profiles make it a viable option for preventing viral infections through nasal administration [124].

**6.2.4 Neoplastic lung diseases:** Lung cancers are divided into non-small cell lung carcinoma (NSCLC – 80%, including squamous cell carcinoma, adenocarcinoma, and large cell carcinoma) and small cell lung carcinoma (SCLC – 20%), according to the World Health Organization (WHO) classification. In the tumor microenvironment, TAMs hamper the activation of cytotoxic T cells and natural killer cells, leading to immune evasion [62]. Recent research has shown advancements in the use of NCs for diagnosing and treating lung cancer, enhancing the efficacy and targeting of cancer therapies.

Natural polymers like gelatin, chitosan [40], and alginate, as well as synthetic polymers such as poloxamer, PLGA [41], and PEG, are widely utilized to develop inhalable nanoformulations. Polymeric micelles have also gained attention for their ability to solubilize hydrophobic drugs, biocompatibility, and increased lung drug retention time [125]. Polymer–drug conjugates can potentially modify the pharmacokinetic profile of drugs delivered to the lungs and enable sustained release. Conjugation of paclitaxel (PTXL) with PEG of different molecular weights prolonged PTXL retention in the lungs and enhanced its antitumor efficacy. Intratracheal administration of these conjugates increased the maximum tolerated dose of PTXL in mice [126].

### 7 Future applications in acute cerebrovascular and traumatic disorders

Neurological disorders encompass a wide range of conditions affecting the nervous system, often resulting from acute injuries such as traumatic brain injury (TBI), strokes, or spinal cord damage. These conditions present significant challenges due to their complex pathophysiology, often involving inflammatory responses.

#### 7.1 Traumatic brain injury (TBI) and stroke

Brain trauma, a major cause of morbidity and mortality, is an acute biomechanical event that disrupts the integrity of brain cells and initiates a series of chronic pathophysiological processes, evolving continuously over time [127]. TBI patients exhibit white matter degradation, protein misfolding, and persistent neuroinflammation. These are accompanied by oxidative stress, production of cerebral cytokines and chemokines, endothelial activation, microglial activation, and the migration of systemic neutrophils, lymphocytes, and monocytes into the injured brain. Such conditions can lead to long-term complications, including physical disabilities, cognitive impairment, and psychiatric disorders [128].

Stroke is the second leading cause of death globally and the third most common cause of disability. TBI might also be a significant risk factor for stroke. Despite the prevalence and significant social and economic impacts of both stroke and TBI, research exploring their connection is limited [129]. Currently, few FDA-approved therapies effectively prevent or treat acute brain injuries, highlighting the need for innovative approaches such as nanomaterials.

The BBB often becomes compromised in these scenarios, allowing for potential therapeutic interventions that would otherwise be hindered. Moreover, microglia exhibit phenotypic similarities to macrophages (usually referred to as M1 and M2 phenotypes), making them a crucial target for NC-based therapies [130–131]. The future of treating these disorders could lie in the precise modulation of these cells using nanomedicine.

#### 7.2 NCs for TBI and stroke treatment

**Figure 2:**
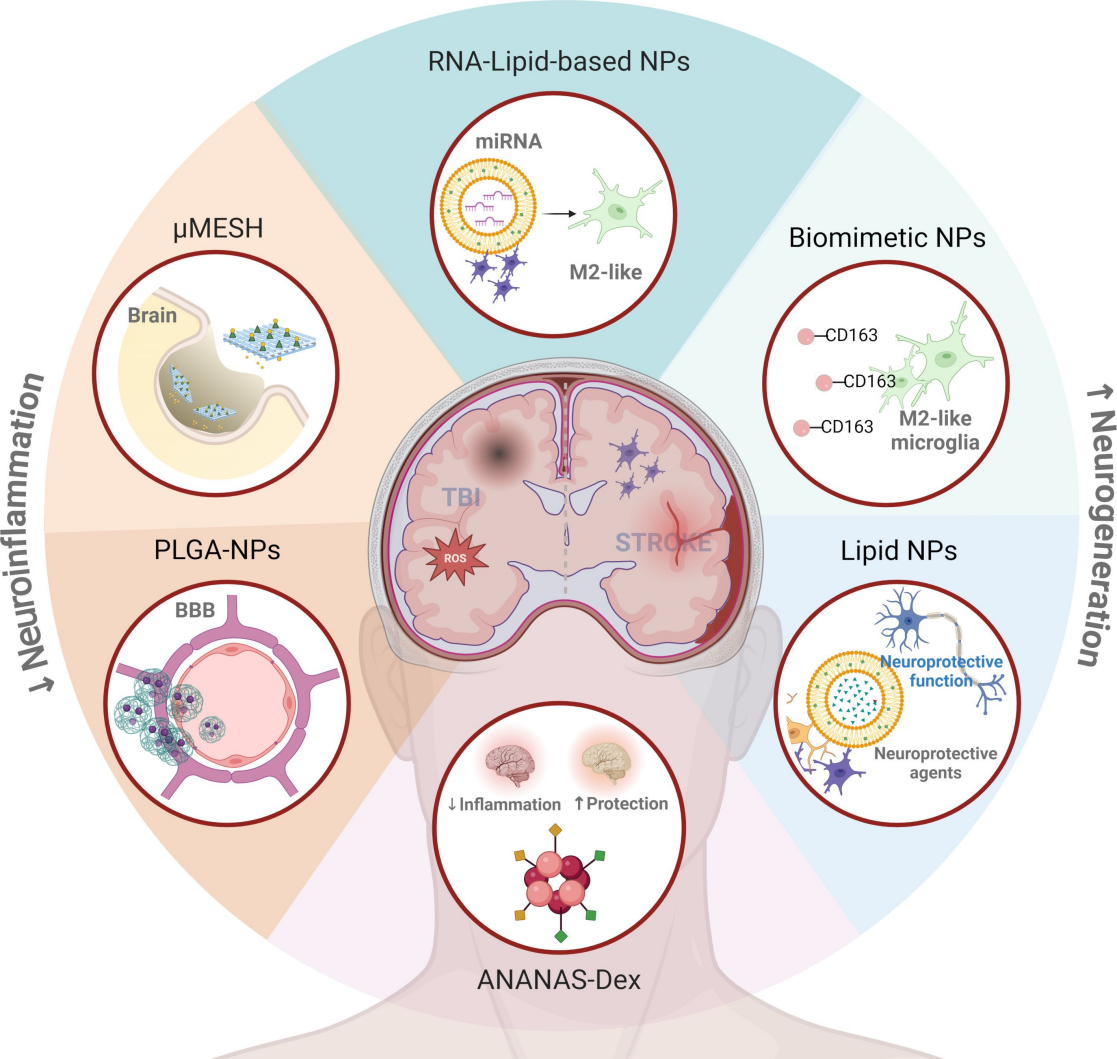
Potential therapeutic strategies in acute cerebrovascular and traumatic disorders. Schematic illustration of therapeutic opportunities designed for mitigating neuroinflammation and achieving neuroprotection and tissue regeneration in TBI and stroke. The nanoformulation may range from simple lipid NPs, encapsulating neuroprotective agents or specific miRNA, to the most complex approaches based on in situ intracranial scaffolds. Created in BioRender. Bigini, P. (2025) https://BioRender.com/f64r030. This content is subject to CC BY 4.0.

NCs can potentially treat stroke and TBI by helping deliver drugs across the BBB and targeting specific cellular pathways involved in neuroinflammation and tissue repair by modulating microglial activity. Figure 2 summarizes the following therapeutic approaches.

**Mitigation of oxidative stress and inflammation:** In TBI, it has been established that neuronal necrosis and parenchymal hemorrhage release significant amounts of free iron into the surrounding tissues, leading to ferroptosis due to excessive oxidative damage to neurons and glial cells [132]. Nanomaterials can deliver antioxidants and anti-inflammatory agents effectively. For instance, polymeric NPs loaded with agents like Dex or Cur have been shown to dampen inflammation and improve neurological outcomes in preclinical TBI models [133–134]. ANANAS, which has been demonstrated to cross the BBB in amyotrophic lateral sclerosis, could potentially benefit TBI treatment by adopting similar mechanisms by delivering anti-inflammatory drugs [135].

Recent research has emphasized the role of ferroptosis, a type of regulated cell death driven by iron-dependent lipid peroxidation, in the TBI. NRF2 is a key regulator of oxidative stress and has been shown to protect against ferroptosis. Compounds like dimethyl fumarate activate NRF2, reducing ferroptosis and neuroinflammation [132]. NCs loaded with NRF2 activators or iron chelators, such as deferoxamine, can mitigate ferroptosis and provide neuroprotection [136].

**Promotion of neuroprotection and tissue regeneration:** In stroke, the priority may be to promote neuroprotection and tissue regeneration, requiring nanomaterials capable of delivering neurotrophic factors or stem cell-derived exosomes. Lipid-based NPs carrying neuroprotective agents can reduce neuronal damage and support brain tissue repair [137]. For instance, mesoporous silica NPs to deliver both miRNA and drugs can modulate macrophage activation states and enhance tissue repair, as demonstrated in various preclinical studies [78,138].

**7.2.1 Enhancing delivery across the blood–brain barrier:** Biocompatibility is essential for nanomaterials, ensuring they do not evoke adverse immune responses. Lipids, polymers, and certain metals have been explored for their compatibility with brain tissue and ability to cross the BBB through active or passive mechanisms. Ensuring that these materials do not cause toxicity or long-term side effects is important for their successful clinical application [139].

Polymeric NPs modified with BBB-penetrating peptides and sialic acid residues enhance brain targeting and drug retention. For instance, loperamide, an opioid drug that typically cannot cross the BBB, was successfully loaded into these modified NCs and administered systemically to rats. The results demonstrated significant central analgesic activity over 24 h, indicating successful brain delivery and retention. Confocal and fluorescent microscopy confirmed brain accumulation, while biodistribution studies revealed that 6% of the injected dose was localized in the CNS for 24 h [140].

In addition to active targeting through surface functionalization, another strategy involves bypassing the BBB via in situ intracranial implants. An example is μMESH, designed for the sustained delivery of chemotherapy drugs such as docetaxel (DTXL) and PTXL. In glioblastoma models, μMESH increased survival from about 30 days (untreated) to 75 days (nanoPTXL-μMESH) and 90 days (PTXL-μMESH). Furthermore, 80% of animals treated with DTXL-μMESH and 60% with nanoDTXL-μMESH were alive after 90 days. These findings suggest that μMESH can effectively deliver chemotherapy to halt aggressive brain tumor growth [141]. These approaches can also be applied to other neurological conditions, such as TBI and stroke, using anti-inflammatory or antioxidant compounds to counteract neuroinflammation and oxidative stress or to promote tissue regeneration.

**7.2.2 Targeting innate immunity in acute brain injuries:** The immune system plays a crucial role in both the progression and resolution of acute brain injuries, such as those caused by TBI and stroke. Similar to KCs in the liver and alveolar macrophages in the lungs, microglia, the resident macrophages of the brain, become aberrantly activated following injury. The primary therapeutic goal in treating brain injuries is to promote regeneration, which involves re-polarizing these microglia and monocyte-derived macrophages towards an anti-inflammatory and pro-resolving state [142]. Nanomaterials designed to cross the BBB, such as gold NPs and liposomes, can deliver neuroprotective agents or growth factors directly to injury sites in the brain. For example, using Lactoferrin-modified PEG-coated polyester NPs can improve neuroprotection in a model of Alzheimer’s disease through intranasal administration [143]. Moreover, 500 nm-diameter NCs made from carboxylated PLGA, an FDA-approved biodegradable biopolymer, have been shown to bind to macrophage receptors and sequester monocytes in the spleen, reducing infiltration of immune cells into the brain and mitigating inflammation, as discussed in previous sections [7].

Biomimetic NPs, such as leukocyte-based NCs, provide stealthiness and evade clearance by the MPS while maintaining delivery capabilities. These NCs have effectively reduced lesion size and inflammation in TBI models [144].

These NPs can modulate the activity of microglia to promote an anti-inflammatory M2 phenotype, facilitating tissue repair and neurogenesis. NPs functionalized with peptides targeting the CD163 receptor on macrophages can direct therapeutic agents to these immune cells, enhancing the resolution of inflammation [60].

**RNA-based therapies:** As highlighted in Section 5.3, RNA-based therapies offer a promising avenue for modulating macrophage and microglia activities. miRNA, siRNA, and mRNA can be delivered via lipid NPs to promote M2 polarization, reduce chronic inflammation, and promote tissue repair. miR-124-3p delivered via lipid NPs has shown potential in promoting M2 polarization in the TBI rat model [145]. Meanwhile, siRNA systems targeting pro-inflammatory pathways can reduce neuroinflammation, such as those developed using rabies virus glycoprotein-derived peptides (RVG-9dR). These systems can specifically target macrophages and microglia via the nicotinic acetylcholine receptor, reducing pro-inflammatory cytokine production and mitigating neuroinflammation [146]. Additionally, mRNA therapies that encode neuroprotective proteins such as IL-10 have demonstrated efficacy in modulating the immune response and supporting recovery in preclinical models of spinal cord injury [147].

Gene therapies delivered via NPs offer another strategy to modulate the immune response post-TBI. For instance, short hairpin RNA-loaded nanodendriplexes targeting chemokine receptors have shown the potential to reduce inflammation and improve the efficacy of stem cell transplantation in TBI models [148]. Melittin-derived cell-permeable peptide (p5RHH) conjugated with mitochondria-targeting miR-146a NPs has been developed to significantly impact the NF-κB pathway, reducing the expression of pro-inflammatory mediators and enhancing neuroprotection post-TBI in controlled cortical impact rat brain models [149].

**Table 3:** Summary of NC application in different pathological conditions and their modes of action.

	Pathological condition	Type of NP	Mode of action	Ref.

Liver	hepatitis	liposomes-PEG-Tenofovir	antiviral effect, improved therapeutic outcomes, and reduced side effects	[85–86]
	hepatitis	galactose-coated NPs-ribavirin	targeting KCs for immunomodulatory effects	[87]
	liver fibrosis	liposomes-valsartan	targeting fibrotic receptor	[90]
	liver fibrosis	liposomes-Dex	M2 polarization	[91]
	liver fibrosis	SPIONs-Ag/ZnO NPs	enhanced drug delivery	[92]
	liver fibrosis	exosome-liposome-clodronate/nintedanib	KCs and pro-fibrogenic activity inhibition	[46]
	liver fibrosis	nanostructured lipid carriers (Cur-mNLCs)	targeting and drug delivery	[94]
	liver fibrosis	β-1,3-ᴅ-glucan-siRNA	macrophage targeting	[95]
	HCC	Fe_3_O_4_ core/Au shell-GBP NPs	photothermal therapy and theranostic applications	[99]
	HCC	lipid-protamine-DNA-PD-L1 NPs	M1 polarization and inflamed cytotoxic T cells	[100]
	AIH	ANANAS-Dex	targeting KCs for immunomodulatory effects	[103]

Lungs	asthma	AuNP-macrophages	immunotheranostic	[112]
	COPD	lipid polymer hybrid NPs	AMs targeting and drug delivery	[113]
	IPF	aeroµGel (nintedanib-PLGA/pirfenidone-liposomes)	AMs evasion and drug delivery	[116]
	SSc-ILD	AuNP-imatinib-CD44	macrophage polarization and drug delivery	[117]
	IPF/ALI	PFC polyplexes-F-PAMD-siRNA	inhibition of macrophage infiltration and lung retention	[118]
	*P. Aeruginosa* infection	M33-nanosystem	lung retention and antimicrobial activity	[122]
	*P. Aeruginosa* infection	microrobots (polymeric NPs-neutrophil membrane-coated-antibiotic)	AMs evasion and antimicrobial activity	[123]
	SARS-CoV-2	ANANAS-hACE2	nanodecoy system	[124]
	NSCC	hyaluronan-based copolymer-CD44-gefitinib/vorinostat	targeting and drug delivery	[125]

Brain	TBI	polymeric-NPs-Dex/Cur	drug delivery	[133–134]
	ALS	ANANAS-Dex	drug delivery	[135]
	stroke	LNPs	neuroprotective agents delivery	[137]
	chronic neuro-diseases	PLGA-Sialic acid-peptide-loperamide NPs	BBB crossing and brain retention	[140]
	glioblastoma	µMESH-DTXL/PTXL	controlled drug release	[141]
	TBI	leukocyte-based NPs	MPS evasion and drug delivery	[144]
	TBI	lipid-miR-124-3p NPs	M2 polarization and neurogenesis	[145]
	TBI	nanodendriplex-RNA	immune response modulation	[148]

## Conclusion

Understanding the behavior of NCs is crucial for revealing their full potential in targeted drug delivery. The interaction of NCs with macrophages, a key component of the MPS, significantly influences therapeutic efficacy. As highlighted in this review, increasing experimental evidence indicates that the innate immune system can profoundly affect the fate of NPs in the body, hindering their therapeutic effectiveness.

Recent advances have shown that modulating the physicochemical properties of NCs, altering administration routes, or damping macrophage activation can help minimize their clearance. This evasion can improve drug accumulation in target tissues, thereby enhancing therapeutic outcomes and reducing side effects. Moreover, leveraging the immune system as a therapeutic target opens new avenues for treating inflammatory disorders.

The application of NCs in liver and lung diseases highlights their versatility and efficacy in targeting specific organs and cells. This success could be translated to brain-related conditions, where NCs can cross the BBB and target neurological disorders, such as TBI and stroke. By harnessing the same principles, NCs can significantly improve therapeutic outcomes in neurological disorders.

The future of NC-based therapies lies in combining multiple methodologies to create multifunctional systems capable of co-delivering anti-inflammatory drugs and growth factors, addressing both immediate inflammatory responses and long-term tissue regeneration needs. This strategy enhances therapeutic outcomes by providing a synergistic effect, targeting different aspects of the pathology, and paving the way for developing theranostic agents. Continued research and innovation in this area are fundamental to fully realizing the potential of nanomedicine and applying these findings to clinical practice, ultimately resulting in enhanced patient outcomes. In this manuscript, we reviewed the main factors influencing the interaction between NCs and macrophages, with a focus on future therapeutic potential. Despite recent advancements, significant challenges remain, particularly the unpredictable nature of NC–macrophage interactions, which can vary by disease state, tissue environment, and NC design. These carriers often accumulate in filter organs like the liver and spleen, reducing their effectiveness in non-hepatic tissue and hindering therapeutic efficacy. It must also address safety concerns, such as hypersensitivity reactions and complement activation from surface modifications like PEGylation. Furthermore, strategies to modulate macrophage activity should balance immune suppression while preserving the body’s natural defenses, as excessive suppression can increase infection risks. Last, achieving scalability, sterility, and consistency in NC production continues to be a significant barrier to clinical translation.

The link between myeloid cells and nanomedicine has become increasingly tight thanks to the growing experimental evidence on NC-mediated biodistribution and accumulation of payloads emerging from preclinical studies.

Interestingly, the uptake of NCs by resident macrophages was first extensively documented in mouse models of neurodegenerative diseases and tumors. In these contexts, in vitro studies showed promising results, such as enhanced brain endothelial transcytosis for BBB passage or selective bioaccumulation in tumors through improved enhanced permeability and retention (EPR) effects, but the translational potential was often undermined in vivo by unexpected filter organ accumulation and active clearance by phagocytic mechanisms [150]. These findings underscore the critical gap between in vitro and in vivo outcomes, highlighting the importance of thoroughly investigating NC fate, including biodistribution and safety profiles, as essential precursors to efficacy studies in specific preclinical models. We should also stress the need to plan preclinical studies with second-generation NCs that can guarantee reliability, repeatability, sterility, stability, and biosafety.

In the last two decades, a plethora of proof-of-concept studies using various nanomaterials have rarely been followed by equally effective translational research. Moreover, current animal models often fail to replicate human pathophysiology or accurately predict NC biodistribution and efficacy, making advancements in this area critical. The vaccination campaign with LNPs for immunization against SARS-CoV-2 could represent an essential turning point moving from the characterization of nanomaterials for biomedical use to the clinical aspect and possible commercialization. “Nanobiology” is now prepared to make a significant leap toward “the next-generation nanomedicine”. The scientific community is urged to make critical decisions to ensure that all efforts and results are not wasted. In this context, we firmly believe that therapies designed to repolarize macrophages could represent a promising and robust area for development.

## Data Availability

Data sharing is not applicable as no new data was generated or analyzed in this study.
